# Long-Term Clinical and Structural Outcomes Following Iris-Claw IOL Exchange for Dislocated Intraocular Lenses

**DOI:** 10.3390/jcm14103306

**Published:** 2025-05-09

**Authors:** Dairis Meiers, Eva Medina, Arturs Zemitis, Juris Vanags, Guna Laganovska

**Affiliations:** 1 Department of Ophthalmology, Riga Stradins University, Dzirciema Street 16, LV-1007 Riga, Latvia; eva.medina@stradini.lv (E.M.); arturs.zemitis@stradini.lv (A.Z.); juris.vanags@stradini.lv (J.V.); guna.laganovska@stradini.lv (G.L.); 2 Pauls Stradins Clinical University Hospital, Pilsonu Street 13, LV-1002 Riga, Latvia

**Keywords:** iris-claw IOL, surgically induced astigmatism, anterior chamber depth, scleral incision, intraocular lens dislocation

## Abstract

**Objectives**: Intraocular lens dislocation is a well-recognized complication of cataract surgery, necessitating secondary interventions such as retropupillary iris-claw IOL implantation. While effective, this procedure requires larger incisions that may induce significant astigmatism. This study aimed to (1) evaluate anterior chamber changes following retropupillary ICIOL implantation and (2) compare surgically induced astigmatism between corneal and scleral incision techniques. **Methods:** In this prospective cohort study, patients with IOL dislocation underwent 25-gauge pars plana vitrectomy with ICIOL implantation. Anterior chamber depth, volume, and angle configuration were measured across 12 meridians preoperatively, at 1–1.5 months (short-term), and 5–6 months (long-term). Surgically induced astigmatism was compared between the corneal and scleral incision groups. Statistical analysis included Shapiro–Wilk, Mann–Whitney U, and repeated-measures ANOVA tests. **Results:** This prospective study included 40 patients (22 females, 18 males) with a mean age of 76.3 ± 5.38 years (range 65–86). Significant reductions in ACD and ACV occurred postoperatively (*p* < 0.05), with partial recovery at long-term follow up. Surgically induced astigmatism was markedly higher with corneal incisions versus scleral approaches (*p* < 0.01 short term; *p* < 0.05 long term). Anterior chamber angle changes varied by meridian but stabilized by 6 months. **Conclusions**: Retropupillary ICIOL implantation induces predictable anterior segment remodeling, with scleral incisions offering superior refractive stability. Surgical planning should prioritize scleral techniques to minimize surgically induced astigmatism while maintaining anatomical efficacy. Future innovations in IOL design may further reduce incision-related complications.

## 1. Introduction

Cataract surgery with intraocular lens (IOL) implantation into the lens capsule is among the most prevalent ophthalmic surgical interventions worldwide [1]. Despite its high success rate, IOL dislocation has emerged as a clinically significant complication, with reported incidence rates ranging from 0.2% to 3% [2,3]. This phenomenon may manifest as either an early or late postoperative event and is frequently associated with predisposing conditions such as pseudoexfoliation syndrome, uveitis, ocular trauma, high myopia, and other pathologies characterized by progressive zonular weakness or capsular instability [4].

The spectrum of IOL dislocation encompasses a continuum of presentations, ranging from mild decentration within the capsular bag to complete luxation into the vitreous cavity or anterior segment, with the degree of displacement correlating with the severity of zonular compromise [5]. While capsular tension rings have demonstrated efficacy in mitigating dislocation risk, they do not provide absolute prevention [6]. Current surgical management strategies include repositioning of the dislocated IOL–capsular complex, IOL exchange, or fixation via iris or scleral suturing techniques, with most cases achieving satisfactory visual outcomes [7,8].

IOL exchange procedures typically involve the explantation of the dislocated lens followed by the implantation of an alternative IOL design, such as an iris-claw IOL (ICIOL) [9]. Originally developed for prepupillary placement to address aphakia while preserving anterior chamber angle (ACA) integrity, early implementations of this technique were associated with significant complications, including endothelial cell loss and iris trauma [10]. Subsequent modifications established the retropupillary implantation technique, wherein the ICIOL is fixated to the posterior iris surface, resulting in a more physiological anatomical position and improved safety profile [11]. This approach has since become a widely adopted solution for aphakia correction in eyes with insufficient capsular support.

While retropupillary ICIOL implantation demonstrates favorable efficacy and safety outcomes compared to transscleral sutured IOLs and angle-supported anterior chamber IOLs, it inherently alters ocular anatomy relative to natural crystalline lens position or in-the-bag IOL placement [12]. The current literature contains limited quantitative data regarding postoperative changes in anterior chamber parameters, particularly depth (ACD), volume (ACV), and angular configuration across different corneal sectors. A comprehensive understanding of these anatomical modifications remains an important area for further investigation [13].

Technical considerations of ICIOL implantation include the requirement for relatively large surgical incisions (typically 5.5–6.0 mm) due to the lens’ rigid design, which frequently results in clinically significant surgically induced astigmatism (SIA) [14]. The magnitude of SIA exhibits dependence on multiple surgical factors, including incision size, location (corneal versus scleral), and architectural configuration (e.g., straight, L-shaped, or corneoscleral tunnel incisions) [15]. Optimization of incision parameters represents a critical factor in achieving optimal refractive outcomes [16].

Despite the established efficacy of retropupillary ICIOL implantation for managing dislocated intraocular lenses, critical knowledge gaps persist regarding its long-term anatomical consequences and optimal surgical approach. While prior studies have demonstrated procedural safety and refractive outcomes, systematic evaluations of anterior chamber architecture—including sectoral changes in ACD, ACV, and angle configuration across 12 ocular sections—remain underexplored. Furthermore, the impact of incision technique (corneal versus scleral) on SIA lacks rigorous comparative analysis in this context. To address these gaps, this study aimed to (1) quantitatively assess anatomical alterations following retropupillary ICIOL implantation, with a particular focus on longitudinal ACD, ACV, and angular changes, and (2) compare scleral and corneal incision techniques to determine their differential effects on postoperative corneal astigmatism and refractive outcomes. By elucidating these relationships, our findings aim to refine surgical protocols, optimize visual rehabilitation, and advance understanding of ICIOL-related biomechanical adaptations.

## 2. Materials and Methods

### 2.1. Study Design

This prospective cohort study was designed to evaluate structural ocular parameter changes following surgical intervention for IOL dislocation.

All patients included in this study underwent a standardized surgical procedure consisting of 25-gauge pars plana vitrectomy with concurrent explantation of the dislocated IOL and implantation of an ICIOL. The surgical approach involved three incisions: two identical corneal paracenteses at the 3 and 9 o’clock positions created using a 15° microvitreoretinal blade and a primary 5.5 mm incision at the 12 o’clock position. Based on the location of this primary incision, patients were systematically divided into two study groups—those receiving a clear corneal tunnel incision (corneal incision group) and those undergoing a scleral tunnel incision with corneal lip (scleral incision group). While this approach is well established, potential limitations include the risk of corneal endothelial cell loss, suture-related complications, induced astigmatism, late dehiscence, and variability in wound healing between techniques.

The sample size was determined by the number of eligible patients treated during the study period, without a prior formal sample size calculation. All procedures were performed by experienced vitreoretinal surgeons using standardized microsurgical equipment and techniques to ensure procedural consistency across cases. Each surgical technique was consistently performed by different operators, and no blinding or masking was applied during the procedures or outcome assessments.

This study was conducted in accordance with the Declaration of Helsinki [17] and received ethical approval from the Rīga Stradiņš University Research Ethics Committee (Decision No. 6-2/6/12, dated 05/09/2019). Written informed consent was obtained from all participants prior to their inclusion in this study.

A comprehensive set of biometric parameters was systematically assessed to quantify the anatomical and refractive consequences of the procedure, including

- Corneal astigmatism;
- ACA measurements in 12 meridians;
- Anterior chamber depth (ACD);
- Anterior chamber volume (ACV).

Standardized ophthalmic evaluations were performed at three time points as follows:

1. Preoperative baseline assessment;
2. Short-term postoperative evaluation (1–1.5 months);
3. Long-term postoperative evaluation (5–6 months).

For analytical purposes, the 1–1.5-month follow-up data were designated as short-term outcomes, while the 5–6-month data were classified as long-term outcomes. This temporal stratification enabled the assessment of both immediate surgical effects and longer-term anatomical adaptations.

### 2.2. Determination of Corneal Astigmatism

Corneal astigmatism was measured using the ANTERION swept-source optical coherence tomographer and biometer (Heidelberg Engineering, Heidelberg, Germany) with its dedicated corneal analysis software (ANTERION CORNEA APP (V.1.2.2)). The system acquires 65 radial scans to generate comprehensive corneal maps and analyses across an 8 mm diameter. Using this standardized protocol, we evaluated multiple corneal parameters including simulated keratometry average, keratometric astigmatism, posterior keratometry average, total corneal power average, TCP astigmatism, central corneal thickness, and corneal diameter.

The total corneal power average represents the cornea’s refractive power calculated through ray tracing across a 3 mm optical zone, accounting for both anterior and posterior corneal curvatures while incorporating the true refractive indices of corneal tissue (1.376) and aqueous humor (1.336). TCP astigmatism was derived as the dioptric difference between the steepest and flattest meridians from these ray-traced calculations, providing a comprehensive assessment of corneal astigmatism that considers the complete optical pathway of both corneal surfaces. This advanced imaging methodology offers precise, reproducible measurements of corneal shape and optical properties critical for refractive analysis.

### 2.3. Determination of ACA, ACD, and ACV

The ANTERION METRICS App was utilized for comprehensive anterior segment evaluation, with the following parameters systematically analyzed: aqueous depth, ACD, ACV, and ACA measurements across 12 clock-hour meridians (ACA_1 through ACA_12). This standardized protocol enabled precise quantification of anterior segment architecture, with all measurements automatically generated by the device’s integrated analysis software to ensure consistency and reproducibility.

### 2.4. Data Analysis

The normality of data distribution was assessed using the Shapiro–Wilk test. Based on the distribution characteristics, appropriate statistical tests were selected for hypothesis testing. For comparisons between two independent groups, the Mann–Whitney U test was employed for non-normally distributed data, while the independent samples Student’s t-test was applied to normally distributed variables. Longitudinal analysis of non-parametric data across multiple time points was performed using the Friedman test, with post hoc pairwise comparisons conducted where appropriate. For normally distributed longitudinal data, repeated measures analysis of variance (ANOVA) was utilized, with Bonferroni correction applied for multiple comparisons when indicated. All statistical analyses were performed using Jamovi software (version 2.6.26) [18], with a predetermined significance threshold of *p* < 0.05 for all tests.

## 3. Results

This study consisted of 40 patients, including 22 females and 18 males. The mean age of the patients was 76.3 ± 5.38 years, with ages ranging from 65 to 86 years.

### 3.1. Corneal Astigmatism

Astigmatism measurements showed significant differences across all incision types, with χ^2^(2) = 44.5 and *p* < 0.001. Post hoc analysis confirmed that astigmatism levels significantly differed between all time points (*p* < 0.001). Before the operation, astigmatism had a median of 0.830 (IQR: 0.565–1.34). In the short term following the operation, the median increased to 3.25 (IQR: 1.55–7.49). However, in the long term after the operation, astigmatism decreased, with a median of 2.395 (IQR: 1.063–4.55).

Astigmatism measurements showed significant differences after a corneal incision, with χ^2^(2) = 46.35 and *p* < 0.001. Post hoc analysis confirmed that astigmatism levels significantly differed between all time points (*p* < 0.001). Before the operation, astigmatism had a median of 0.730 (IQR: 0.520–1.35). In the short term following the operation, the median increased to 5.23 (IQR: 3.115–8.58). However, in the long term after the operation, astigmatism decreased, with a median of 3.43 (IQR: 1.755–5.96).

No statistically significant difference in astigmatism was observed between the groups following the scleral incision procedure. Astigmatism did not differ between the groups prior to the operation.

In the short term after the operation, astigmatism was noticeably higher following a corneal incision (6.01 ± 3.66) compared to a scleral incision (1.42 ± 0.872). This difference was statistically significant, with an average mean difference of 4.59, 95% TI [2.49–6.69], t (38.0) = 4.43, *p* < 0.001, and d = 1.5.

In the long term after the operation, astigmatism showed a statistically significant difference between the corneal group (median = 3.43, IQR: 1.755–5.96) and the scleral group (median = 1.09, IQR 0.830–1.75), U = 59.0, *p* < 0.001, and r = 0.664.

### 3.2. ACD, ACV, and ACA

A subgroup analysis was conducted within the cohort that underwent corneal incision, focusing on changes in ACD, ACV, and ACA at all clock-hour positions.

ACD showed a statistically significant difference, F (2, 48) = 45.7, *p* < 0.001, and **η_G_^2^** = 0.420. Tukey’s post hoc analysis revealed that ACD before surgery was significantly different from short-term measurements after surgery, with a mean difference of 0.866; *p* < 0.001. Additionally, ACD before surgery and long-term post-surgery measurements were significantly different, with a mean difference of 1.042; *p* < 0.001. Short-term post-surgery ACD was also significantly different from long-term post-surgery values, with a mean difference of 0.176; *p* = 0.018.

ACV showed a statistically significant difference, F (2, 50) = 16.3, *p* < 0.001, and η_G_^2^ = 0.026. Tukey’s post hoc analysis revealed that ACV before surgery was significantly different from short-term measurements after surgery, with a mean difference of 12.7; *p* < 0.001. Additionally, ACD before surgery and long-term post-surgery measurements were significantly different, with a mean difference of 9.67; *p* = 0.002. The changes in ACA are presented in Table 1.

Tukey’s post hoc analysis demonstrated that ACA at various clock hours before surgery exhibited significant differences compared to short-term and long-term postoperative measurements. Specifically, ACA at 1 o’clock prior to surgery was significantly different from short-term postoperative measurements, with a mean difference of 3.46; *p* = 0.024. Similarly, ACA at 3 o’clock before surgery showed a significant difference from short-term postoperative measurements, with a mean difference of −5.269; *p* = 0.004.

At 4 o’clock, ACA before surgery was significantly different from both short-term postoperative measurements, with a mean difference = −7.62 with *p* = 0.009, and long-term postoperative measurements, with a mean difference = 2.31 with *p* = 0.02. Likewise, ACA at 9 o’clock prior to surgery was significantly different from short-term postoperative values, with a mean difference = −7.923 with *p* = 0.009, and it also differed significantly from long-term postoperative measurements, with a mean difference = −7.231 with *p* = 0.043.

Although ACA at 10 o’clock exhibited statistical significance, Tukey’s post hoc analysis did not identify any specific differences. Finally, ACA at 12 o’clock prior to surgery was significantly different from short-term postoperative measurements, with a mean difference of 4.15 with *p* = 0.028.

## 4. Discussion

The present study systematically evaluated the anatomical and refractive outcomes following retropupillary ICIOL implantation, with particular emphasis on the quantitative assessment of anterior segment parameters. Our investigation focused on longitudinal changes in ACD, ACV, and angular configuration across 12 meridional sections while concurrently comparing the refractive implications of corneal versus scleral incision approaches. The results provide substantive evidence regarding (1) the dynamic structural adaptations of the anterior segment post-ICIOL implantation and (2) the differential effects of surgical technique on induced astigmatism and visual outcomes. These findings offer clinically relevant insights for optimizing the surgical management of aphakia in eyes with compromised capsular support, addressing critical gaps in the existing literature.

### 4.1. Anatomical Changes in the Anterior Chamber

The observed alterations in ACD and ACV following ICIOL implantation are consistent with previous findings by Gicquel et al. [19] and Mohr et al. [20], confirming that secondary IOL procedures induce significant anatomical modifications. The characteristic postoperative reduction in both ACD and ACV primarily results from the posterior fixation mechanism of the ICIOL, which fundamentally alters the anterior–posterior ocular architecture compared to conventional in-the-bag IOL placement [21]. While our data revealed an immediate, pronounced decrease in ACD and ACV during the early postoperative period (1–1.5 months), subsequent measurements (5–6 months) demonstrated partial recovery, suggesting gradual anatomical adaptation. The statistically significant differences in ACD between preoperative values and both postoperative intervals (*p* < 0.05) indicate persistent structural remodeling following ICIOL fixation. These findings have important clinical implications, particularly regarding the need for comprehensive preoperative assessment to account for potential alterations in anterior segment dynamics that may affect intraocular pressure regulation and long-term ocular homeostasis.

### 4.2. SIA

The induction of postoperative astigmatism represents a clinically significant determinant of visual outcomes following ICIOL implantation. Clear corneal incisions on the steep axis during phacoemulsification reduce pre-existing astigmatism (0.96 D to 0.78 D, *p* ≤ 0.05), while adding opposite clear corneal incisions significantly enhances this effect (2.23 D to 1.56 D, *p* ≤ 0.05), demonstrating a safe and effective method for astigmatism correction in cataract surgery [22]. Our findings demonstrate a pronounced increase in median astigmatism during the early postoperative period, attributable to the requisite large surgical incisions for rigid ICIOL implantation. Quantitative analysis revealed substantially greater SIA in cases employing corneal incisions compared to scleral approaches, with this difference maintaining statistical significance at both short-term (1–1.5 months; *p* < 0.01) and long-term (5–6 months; *p* < 0.05) follow-up intervals. These observations align with previous investigations by Kwon et al. [23] and Gonnermann et al. [24], who similarly reported increased SIA associated with anterior corneal incisions—a phenomenon attributed to their anatomical proximity to the visual axis and consequent disruption of corneal asphericity. The persistent nature of these astigmatic changes underscores the importance of meticulous surgical planning, particularly regarding incision location and architecture, to optimize refractive outcomes in ICIOL procedures [25].

In contrast, scleral incisions demonstrated significantly lower SIA compared to corneal approaches (*p* < 0.05). This refractive advantage likely stems from two principal factors: (1) the more peripheral location of scleral incisions, which preserves central corneal topography, and (2) their reduced biomechanical impact on corneal structural integrity. The statistically significant difference in SIA between techniques persisted throughout all postoperative follow-up periods (*p* < 0.01 at 1.5 months; *p* < 0.05 at 6 months), highlighting incision location as a critical determinant of refractive stability. These findings support the preferential use of scleral incisions, which align with current surgical paradigms advocating peripheral, small-incision techniques to maximize postoperative visual outcomes [26].

### 4.3. Changes in ACAs

The exchange of dislocated intraocular lenses (IOLs) led to significant alterations in ACA, with regional variations observed across different clock-hour positions. Our results demonstrated a transient decrease in ACA at the 12 and 1 o’clock positions postoperatively, followed by a tendency to return toward baseline, while angles at 3, 4, 9, and 10 o’clock showed a sustained increase in both short- and long-term follow ups. These findings align with previous studies, such as Hayashi et al. [27], who reported a significant postoperative widening of ACA in patients with primary angle-closure glaucoma (PACG), correlating with improved aqueous outflow and reduced intraocular pressure (IOP).

Furthermore, Kim et al. [28] found that phacoemulsification with IOL implantation resulted in a marked deepening of the anterior chamber and increased ACA width, particularly benefiting patients with narrow angles by reducing the risk of angle-closure glaucoma. Our study similarly observed a long-term increase in ACA at multiple positions, suggesting that IOL repositioning may alleviate anterior segment crowding and facilitate improved aqueous drainage. However, the transient narrowing observed at 12 and 1 o’clock may indicate localized anatomical or biomechanical responses to surgical intervention, warranting closer postoperative monitoring to mitigate potential risks of aqueous outflow obstruction.

These findings underscore the critical need for personalized preoperative assessment in patients requiring IOL exchange, especially those with pre-existing risk factors for glaucoma (e.g., pseudoexfoliation syndrome, narrow angles, or elevated IOP). Eyes with pseudoexfoliation syndrome exhibit reduced ACD, ACV, and axial length [29]. The observed sectoral variations in ACA configuration suggest that advanced imaging modalities—particularly anterior segment optical coherence tomography—could enhance surgical planning by

1. Providing high-resolution, cross-sectional visualization of angle anatomy;
2. Quantifying localized areas of angle narrowing that may not be apparent on clinical examination;
3. Enabling precise postoperative monitoring of angle configuration changes.

Such detailed anatomical characterization may help optimize IOL selection and positioning while mitigating potential postoperative pressure complications in at-risk eyes. Future studies incorporating anterior segment optical coherence tomography-based angle metrics could further refine risk stratification protocols for these procedures.

### 4.4. Clinical Implications and Surgical Technique Considerations

While ICIOL implantation provides an anatomically stable and clinically effective solution for aphakia, the necessity for larger incisions presents a significant refractive trade-off through increased risk of SIA. Iris fixation of late dislocated IOL–capsular bag complexes provides superior visual outcomes, less astigmatism, better IOP control, and fewer complications (including CME and endothelial cell loss) compared to anterior chamber IOL implantation [30]. Suture fixation of IOLs in eyes with inadequate capsular support is a safe and effective procedure, offering stable refractive outcomes (−0.375 D mean deviation from target), low complication rates (6.3% tilt, 7.2% decentration), and reduced dependence on refractive aids, with pseudoexfoliation syndrome (33%), trauma (17%), and prior complicated cataract surgery (11%) being the most common indications [31]. Our findings confirm that both incision size and location serve as critical modifiable factors influencing SIA, underscoring the importance of adopting surgical strategies that prioritize (1) minimization of incision dimensions and (2) utilization of scleral or scleral-tunnel techniques to optimize refractive outcomes.

Longitudinal analysis revealed a partial reduction in astigmatism over time, though values remained elevated compared to preoperative baselines (*p* < 0.05 at 6 months). This pattern suggests some degree of corneal compensatory remodeling following incision-induced changes, though the variability observed between cases emphasizes the necessity for individualized surgical planning and postoperative management. These results align with long-term follow-up studies by Gicquel et al. [19] and Forlini et al. [32], who similarly demonstrated that careful consideration of incision parameters—particularly size and location—represents a key determinant in minimizing astigmatic changes and achieving optimal visual outcomes.

While this study provides clinically relevant insights, we acknowledge that the sample size of 40 patients, though determined by the available cohort during the study period, may limit statistical power. The relatively small number particularly affects subgroup comparisons between surgical techniques. However, our standardized surgical protocols and single-center design helped maintain procedural consistency. Future multicenter studies with larger cohorts would help validate these preliminary findings.

Future research should prioritize several key directions to advance ICIOL outcomes: (1) the development of foldable IOL materials compatible with sub-3 mm microincisions to minimize astigmatic burden [33]; (2) quantitative analysis of corneal biomechanical changes using advanced imaging modalities, like Scheimpflug tomography or OCT-based strain mapping [34]; (3) comparative studies evaluating refractive predictability of sutureless scleral fixation techniques (e.g., glued IOL vs. Yamane) [35]; and (4) the implementation of machine learning algorithms to preoperatively predict SIA based on incision parameters, corneal characteristics, and patient demographics [36]. Together, these innovations would bridge the gap between structural stability and refractive precision, ultimately enhancing both surgical outcomes and patient satisfaction.

## 5. Conclusions

Our study confirms that retropupillary ICIOL implantation induces significant anterior segment changes, with corneal incisions generating higher SIA than scleral approaches (*p* < 0.05). To optimize refractive outcomes, scleral or scleral tunnel incisions should be prioritized when anatomically feasible. While postoperative corneal remodeling partially offsets early SIA, its persistence underscores the need for innovation in IOL design and microincisional techniques to minimize incision-related effects. Future work should focus on lens designs compatible with smaller incisions, balancing structural stability and refractive precision to improve patient outcomes.

## Figures and Tables

**Table 1:** Summary of the changes in ACA.

	Before	ST After	LT After	df	F	*p*-Value	η_G_^2^
**1 o’clock (mean ± SD)**	**46.8 ± 9.15**	**43.4 ± 8.99**	**45.3 ± 9.3**	**(2, 50)**	**3.24**	**0.048**	**0.024**
2 o’clock (mean ± SD)	48.6 ± 8.99	50.0 ± 7.75	50.7 ± 9.73	(2, 50)	0.967	0.387	0.01
**3 o’clock (mean ± SD)**	**50.7 ± 9.85**	**56.0 ± 9.72**	**55.4 ± 10.7**	**(2, 50)**	**7.29**	**0.002**	**0.054**
**4 o’clock (mean ± SD)**	**49.1 ± 12.1**	**56.7 ± 9.86**	**54.4 ± 10.4**	**(2, 50)**	**6.82**	**0.002**	**0.083**
5 o’clock (mean ± SD)	48.3 ± 8.81	50.0 ± 8.72	49.4 ± 8.49	(2, 50)	0.566	0.571	0.007
6 o’clock (mean ± SD)	47.2 ± 9.54	47.5 ± 8.62	49.5 ± 10.6	(2, 50)	1.01	0.372	0.012
7 o’clock (mean ± SD)	48.1 ± 10.3	50.7 ± 9.74	51.3 ± 10.3	(2, 50)	2.01	0.145	0.019
8 o’clock (mean ± SD)	51.8 ± 11.5	54.8 ± 9.09	55.2 ± 9.83	(2, 50)	1.39	0.259	0.022
**9 o’clock (mean ± SD)**	**47.0 ± 11.2**	**54.9 ± 11.5**	**54.2 ± 11.5**	**(2, 50)**	**7.24**	**0.002**	**0.093**
**10 o’clock (mean ± SD)**	**46.0 ± 11.0**	**50.1 ± 8.26**	**49.6 ± 8.76**	**(2, 50)**	**3.3**	**0.045**	**0.038**
11 o’clock (mean ± SD)	45.7 ± 10.1	42.4 ± 8.31	43.7 ± 9.42	(2, 50)	2.23	0.118	0.021
**12 o’clock (mean ± SD)**	**44.3 ± 9.74**	**40.1 ± 10.4**	**42.0 ± 11.9**	**(2, 50)**	**3.91**	**0.026**	**0.026**

df = degrees of freedom; F = F-statistic; *p* = probability value; η_G_^2^ = partial eta-squared (effect size). Statistically significant o’clock position values are in bold.

## Data Availability

The data used to support the findings of this study are available from the corresponding author upon reasonable request.

## Abbreviations

The following abbreviations are used in this manuscript:

IOL	intraocular lens
ICIOL	iris-claw intraocular lens
ACD	anterior chamber depth
ACV	anterior chamber volume
SIA	surgically induced astigmatism
ACA	anterior chamber angle
